# Incidental Discovery of a Membranous Ventricular Septal Aneurysm in Two Dissimilar Patients

**DOI:** 10.1155/2012/324326

**Published:** 2012-09-18

**Authors:** Abhishek Naidu, Michelle Ricketts, Aashish Goela, Gerard Shoemaker, Shuo Li

**Affiliations:** ^1^Schulich School of Medicine & Dentistry, The University of Western Ontario, London, ON, Canada N6A 5C1; ^2^Department of Medical Imaging, The University of Western Ontario, London, ON, Canada N6A 5W9; ^3^London Health Sciences Centre—University Hospital, The University of Western Ontario, London, ON, Canada N6A 5A5; ^4^The University of Western Ontario, London, ON, Canada N6A 3K7; ^5^GE Healthcare, Mississauga, ON, Canada L5N 5P9

## Abstract

A ventricular septal aneurysm (VSA) is a rare cardiac anomaly, and an accurate statistic of its prevalence has not been reported in the literature. True incidence is likely underestimated as most patients are thought to be asymptomatic. As a result, most VSAs are discovered incidentally on echocardiography, during angiography, or at autopsy. Potential complications include rupture, bacterial endocarditis, right ventricular outflow tract obstruction, and thromboembolic disease. It has been proposed that VSAs occur in association with ventricular septal defects (VSDs) and other congenital cardiac abnormalities. It is uncommon for a VSA to exist in the absence of a known prior ventricular septal defect. We present two cases, each highlighting an incidental intact aneurysm involving the membranous interventricular septum. We discuss the contrast in the two patients with regard to their age, accompanying cardiac anomalies and cardiovascular fitness. Clinical implications of the condition are reviewed.

## 1. Introduction 

An aneurysm of the membranous interventricular septum is an uncommon cardiac abnormality with no incidence statistic reported in the literature. It has been documented in isolation but is seen in association with congenital heart disease in 0.3% of cases and in association with VSDS in 19% of cases [1, 2]. Etiologies of the condition include idiopathic formation, trauma, infection, or as the result of spontaneous closure of a pre-existing VSD [2–6]. The majority of cases are thought to be congenital in origin [4, 6].

The rarity and obscurity of its clinical symptoms and the potential sequelae of membranous VSAs attest to the importance of awareness among clinicians and radiologists. Clinical manifestations are variable. Some patients are asymptomatic with normal physical examination whereas others can present with hemodynamic compromise [6]. Possible complications include conduction abnormalities, aneurysm rupture, bacterial endocarditis, right ventricular outflow tract obstruction, significant intracardiac shunting, and thromboembolism. Herein, we report two cases of an aneurysm of the membranous ventricular septum detected incidentally during echocardiography and confirmed on computed tomography (CT). 

## 2. Case One 

**Figure 1:**
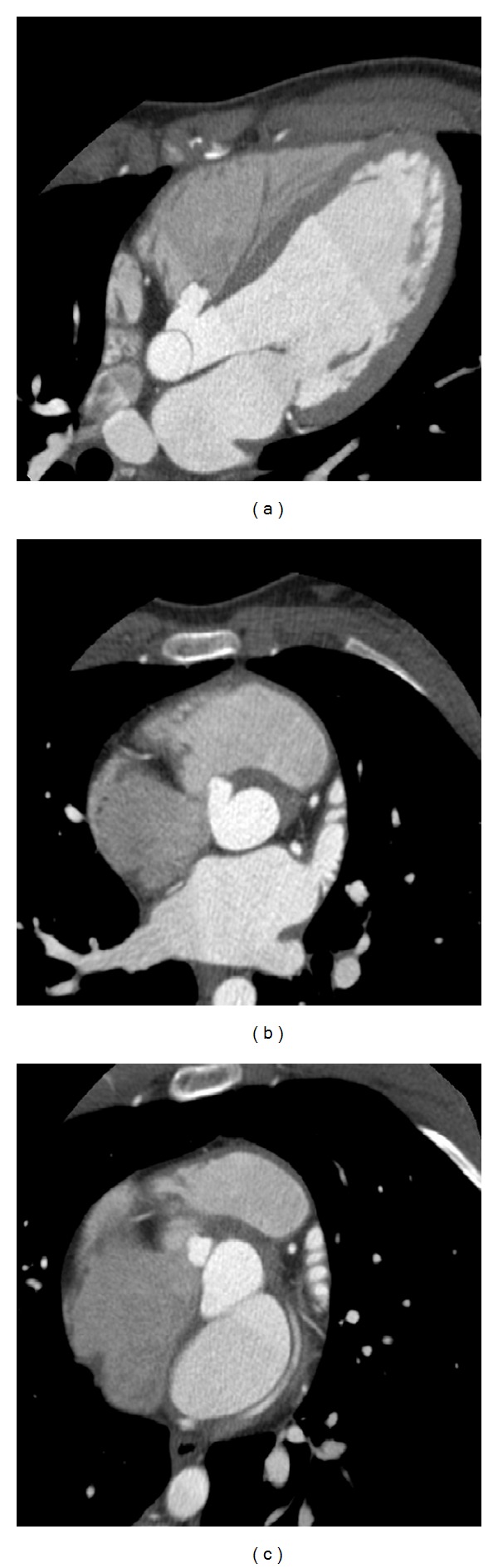
CT imaging results of the female track and field athlete. (a) Four-chamber view depicting an aneurysm of the membranous ventricular septum. (b)-(c) Short axis views illustrating the aneurysm.

An 18-year-old female track and field athlete with bradycardia was referred to a cardiologist by her family physician to rule out a cardiac defect. She underwent a 2D echocardiogram, which revealed what was thought to represent a poorly visualized aneurysm. More specifically, the abnormality was initially felt to be an aneurysm of a sinus of Valsalva; though, the lack of certainty required further imaging. A prospectively gated contrast enhanced multidetector CT (MDCT) scan of the heart was performed. CT imaging (Figure 1) confirmed the presence of an aneurysm; however, it was located at the membranous interventricular septum immediately caudal to the level of the right coronary sinus. The aneurysm measured 1.2 × 0.9 cm, stretching the septum into the right ventricle. No accompanying VSD was evident.

Following the diagnosis, the patient remained asymptomatic, but persistently bradycardic. She denied any angina upon exertion (Canadian Cardiovascular Society class 0), allowing for her status as an elite track and field athlete, typically under cardiovascular stress. The patient denied symptoms of exertional dyspnea, orthopnea, paroxysmal nocturnal dyspnea, or ankle edema (New York Heart Association class I). There were no reports of palpitations or any other respiratory or cardiovascular symptoms. However, the patient recounted a history of 3 syncopal episodes since age 11, previously felt to reflect vasovagal syncope rather than manifestations of underlying structural heart disease. During physical examination, the patient had a regular heart rate of 45 beats per minute and blood pressures in a seated position measuring 95/65 mmHg and 100/65 mmHg in the left and right arm, respectively. Her right lower limb systolic blood pressure was measured at 110 mmHg. On auscultation, grade 1 systolic ejection and pansystolic murmurs were heard along the left sternal border. According to the patient's mother, heart murmurs were noted at birth, but no echocardiogram was performed at the time. Jugular venous pressure and carotid upstroke remained within normal limits, and no breathing abnormalities were identified. A transesophageal echocardiogram (TEE) was ordered to assess the resiliency of her aneurysm, which was subsequently found to be intact. There was no evidence of intracardiac shunting, right ventricular hypertrophy, pulmonary hypertension, or any other haemodynamic abnormalities. Essentially, the patient was entirely asymptomatic from a cardiac perspective. 

## 3. Case Two 

**Figure 2:**
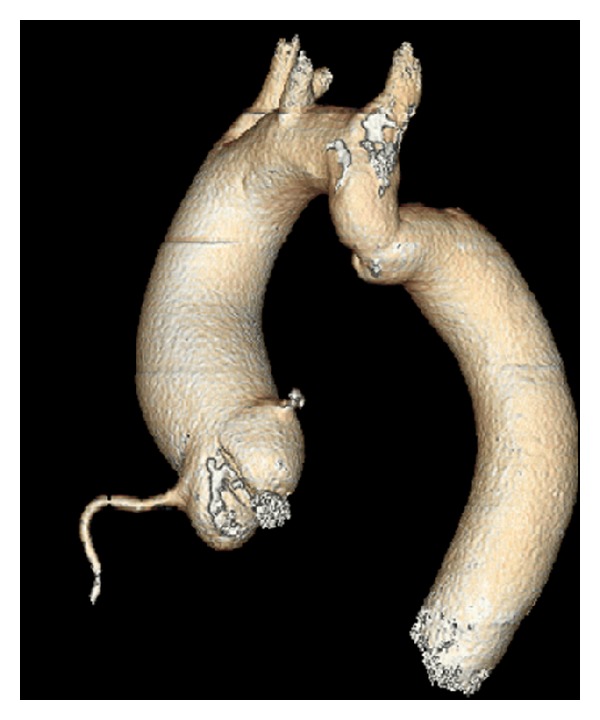
Three-dimensional volume-rendered image of the aorta in the elderly patient demonstrating mild aortic coarctation distal to the left subclavian artery.

**Figure 3:**
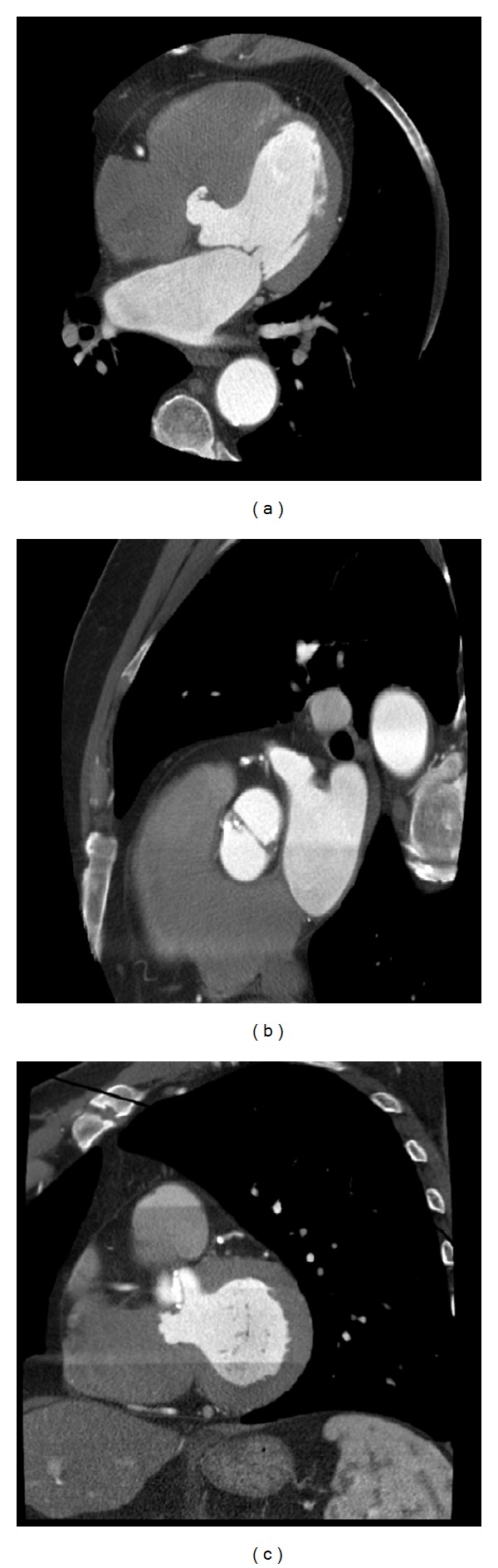
CT imaging results of the elderly patient (a) four-chamber view evidencing a membranous ventricular septal aneurysm. (b)-(c) Short axis views depicting the aneurysm.

A 60-year-old man complaining of exertional dyspnea, palpitations, and fatigue upon slight physical activity (New York Heart Association class 3) underwent cardiac referral for further assessment. A TEE revealed a calcified bicuspid aortic valve with severe aortic valve stenosis (aortic valve area measured at 0.8 cm^2^). A poorly visualized abnormality was seen involving the interventricular septum, and a cardiac CT was arranged for further characterization. CT imaging demonstrated mild aortic coarctation distal to the left subclavian artery (Figure 2) as well as an aneurysm of the membranous interventricular septum without an associated VSD (Figure 3). The aneurysm, measuring 2.2 cm ×1.7 cm ×1.6 cm, projected into the right ventricle. 

The patient was referred to cardiac surgery, and informed consent was obtained for aortic valve replacement. A second TEE completed prior to surgery revealed the absence of intracardiac shunting or a VSD. An aortotomy was performed 3 mm above the right coronary artery, and traction sutures were positioned to expose the aortic valve. The aneurysm of the ventricular septum was subsequently identified intraoperatively, and further inspection of the ventricular septum ruled out a VSD. The decision was made to not intervene with regards to the aneurysm to prevent potential disruption of the patient's conduction system, thereby minimizing the chance of post-operative arrhythmia. 

## 4. Discussion 

Aneurysms of the membranous ventricular septum are extremely rare with very few case reports in the literature. An association with other cardiac defects has been acknowledged. In particular, VSAs have been found to occur in association with VSDs in 19% of cases. VSAs have also been reported in isolation without an accompanying VSD, as demonstrated by our two cases; however, this is a rare manifestation and is usually diagnosed incidentally [2, 5]. 

The first case is an example of an isolated VSA discovered incidentally in a young athlete with a very active lifestyle. The only relevant findings on physical examination were mild grade I murmurs. Despite its rarity, a comprehensive differential diagnosis of a congenital heart murmur presenting in adolescence could include an aneurysm of the membranous interventricular septum. A study has suggested that a late systolic murmur heard primarily along the left sternal border could signify the existence of a VSA [7]. 

Although patients are typically asymptomatic, an isolated VSA is not without potential risks. Such a condition requires careful diagnosis and analysis, as rupture, bacterial endocarditis, right ventricular outflow tract obstruction, intracardiac shunting, and thromboembolism, among other cardiac complications can occur as a result of a membranous VSA. Its presence in an asymptomatic elite track and field athlete is particularly unique. Concerns arose regarding the patient's continued participation in athletics. Without the presence of right ventricular hypertrophy, intracardiac shunting, pulmonary hypertension, haemodynamic abnormalities, or other cardiac complications, the prognosis for the 18-year-old athlete was felt to be favourable, and her continued involvement in competitive athletics was not prohibited. According to eligibility recommendations for competitive athletes with cardiac conditions, enacted by the American College of Cardiology, the patient did not possess the appropriate contraindications, such as hemodynamic compromise, for her participation in competitive sports [8]. She receives routine echocardiogram monitoring to assess the VSA. 

The second case discusses a patient with a VSA discovered incidentally during workup for symptomatic aortic stenosis secondary to a sclerotic bicuspid valve. It has been reported that an aneurysm of the membranous ventricular septum coexists with congenital heart disease in 0.3% of patients. In addition to a VSA, our patient possessed both a bicuspid aortic valve and mild aortic coarctation. Yavuz et al. described a 22-year-old male with a calcified bicuspid aortic valve who was incidentally diagnosed with a VSA during elective surgery [2]. Future attention to this potential lesion and identifying its associated cardiac anomalies could be helpful in establishing an association. 

The second case also demonstrates the role of surgical repair in patients with an asymptomatic VSA. Although aortic valve replacement was the intended surgical intervention, the discovery of the VSA raised the dilemma of performing concomitant aneurysm repair. Among the multiple structural cardiac abnormalities, it was felt that severe aortic stenosis was likely most contributory to the patient's symptoms. As such, isolated aortic valve replacement was performed. Further, the aneurysm was not surgically altered due to the potential for an iatrogenic conduction block. The aneurysm was felt to be benign, posing no immediate risk to the patient, and was not felt to be contributing to the patient's symptoms. Surgical correction of a VSA may be required when haemodynamic abnormalities and other aneurysm-related complications are evident [2]. Conversely, a report by Yilmaz et al. recommended that surgical intervention should be conducted to prevent further enlargement and potential sequelae of a membranous VSA even in the absence of cardiac symptoms [3]. 

These novel case reports address the management of isolated VSAs in both a young healthy athlete and in an older patient undergoing surgical repair for an alternate structural abnormality. Additionally, our second case elucidates the possible association between a bicuspid valve and the manifestation of a VSA. Incidentally diagnosed membranous VSAs should prompt investigations for relevant cardiac abnormalities and further diagnostic evaluation [1]. Interestingly, both cases attest to conservative management of the condition as being the most pragmatic. Nevertheless, careful outpatient monitoring must be conducted. Patients should be cautioned and educated about potential complications that may arise and their indicative symptoms.
